# Impact of Stem Design on Periprosthetic Femoral Fracture Risk: Findings from 182,118 Primary Total Hip Arthroplasties in the Swiss National Joint Registry

**DOI:** 10.2106/JBJS.25.01203

**Published:** 2026-02-19

**Authors:** P.A. Bouché, A. Lübbeke, C. Brand, A.I. Gonzalez, M. Beck, D. Hannouche

**Affiliations:** 1 Division of Orthopedic Surgery and Traumatology, Geneva University Hospitals, Geneva, Switzerland; 2 Faculty of Medicine, University of Paris Cité, Paris, France; 3 Faculty of Medicine, University of Geneva, Geneva, Switzerland; 4 Nuffield Department of Orthopaedics, Rheumatology and Musculoskeletal Sciences, University of Oxford, Oxford, United Kingdom; 5 Swiss National Joint Registry (SIRIS), Bern, Switzerland; 6 Institute of Social and Preventive Medicine, Bern, Switzerland; 7 Orthopaedic Clinic Lucerne, Lucerne, Switzerland

## Abstract

**Background:**

Periprosthetic femoral fractures (PFFs) are an increasingly common indication for revision total hip arthroplasty (THA). While patient-related risk factors are well documented, the influence of femoral stem design on PFF risk remains poorly characterized. In this study using nationwide data, we assessed the association between stem design and PFF risk.

**Methods:**

We analyzed 182,118 primary THAs (performed from 2015 to 2023) from the Swiss National Joint Registry (SIRIS). Cementless stems were categorized according to the Kheir classification, and cemented stems were categorized as double-tapered polished, triple-tapered polished, composite-beam, or custom. A multivariable Cox regression model, including variables such as age, sex, American Society of Anesthesiologists (ASA) class, body mass index (BMI), surgical indication, prior ipsilateral hip surgery, stem design, collar, dual-mobility cup, bearing, and head size, was analyzed. Hazard ratios (HRs) with 95% confidence intervals (CIs) are reported.

**Results:**

Among 182,118 THAs (mean patient age, 68.9 ± 11.5 years; female sex in 53.1% of cases), 1,226 (0.7%) were complicated by PFF. The cumulative incidence of PFF reached 0.7% at 5 years and 1.3% at 10 years. Higher PFF risk was associated with an age of 75 to 84 years (HR = 1.68 [95% CI = 1.44 to 1.96]) and ≥85 years (HR = 1.86 [95% CI = 1.47 to 2.35]), ASA class of 3 to 5 (females, HR = 1.70; males, HR = 1.73), BMI of <18.5 kg/m^2^ (HR = 1.61) or ≥40 kg/m^2^ (HR = 1.64), prior ipsilateral hip surgery (HR = 1.32), and use of a dual-mobility cup (HR = 1.56). Elective procedures (HR = 0.36) and collared stems (HR = 0.26) were associated with a lower risk. Compared with cementless type-3 stems, cementless type-7 (anatomic) stems showed a higher risk of PFF (HR = 1.88), whereas cementless type-1B (HR = 0.62) and composite-beam cemented stems (HR = 0.45) were protective.

**Conclusions:**

In this large nationwide registry study, femoral stem design independently influenced PFF risk after primary THA. Cementless anatomic stems increased the risk, whereas composite-beam cemented stems and the presence of a collar conferred a protective effect. These findings support personalized implant selection, particularly for older patients or those with frailty.

**Level of Evidence:**

Prognostic Level III. See Instructions for Authors for a complete description of levels of evidence.

Among contemporary surgical interventions, total hip arthroplasty (THA) occupies a central role in orthopaedic practice because of its proven efficacy in restoring function and alleviating pain in patients with advanced hip disease^1,2^. Rising life expectancy and the prevalence of degenerative joint conditions have increased the numbers of both primary THA procedures and subsequent revisions^3^.

Recent advances in implant materials and design^4^ have contributed to a decline in revisions due to aseptic loosening^5-8^. Likewise, the use of dual-mobility constructs, particularly in patients at higher risk for dislocation, has substantially reduced the incidence of dislocation-related failure of THA^9^. In this changing context, periprosthetic femoral fractures (PFFs) have emerged as a major cause of revision, now approaching periprosthetic joint infection in clinical importance^10,11^.

Despite the increasing proportion of these fractures, PFFs remain insufficiently characterized. Intraoperative PFFs are partially explained by well-established risk factors, such as the surgical approach^12^. In the present study, we chose to focus specifically on postoperative PFFs and, in particular, to investigate the role of femoral stem design^13^ in their occurrence. Prior studies have suggested that cemented stems—notably those with a composite-beam geometry—may confer a protective effect compared with cementless stems^14,15^. Patient-related factors, including advanced age, female sex, rheumatoid arthritis, and substance use disorders, have also been associated with increased PFF risk^16-18^. More recently, attention has turned to design features such as the presence of a collar, which may reduce the risk of postoperative fracture^19^.

While several studies have examined the association between stem design and PFF, few have provided comprehensive comparisons across all femoral stem types. The aim of the current study was to evaluate the relationship between femoral stem design and the incidence of postoperative PFF using prospectively collected data from the Swiss National Joint Registry (SIRIS), a nationwide database capturing all joint replacement procedures in Switzerland.

## Materials and Methods

This registry-based prospective cohort study was approved by the local ethics committee, and all patients provided written informed consent to participate in the registry.

### Data Sources

Data were obtained from SIRIS, a comprehensive national registry that has prospectively collected information on all THA procedures performed in Switzerland since 2012. With data from both public and private hospitals, the registry has achieved coverage rates of 98.68% of primary hip procedures and >98% of revision hip procedures^20,21^.

### Participants

Patients who underwent primary THA between January 1, 2015, and December 31, 2023, were identified from the SIRIS database. Among the 182,118 eligible primary THAs, 1,226 (0.7%) were complicated by a PFF. Follow-up was censored in December 2024. The mean patient age (and standard deviation) was 68.9 ± 11.5 years (range, 12 to 108 years); in 53.1% (96,669) of the 182,118 cases, the patient was female. The mean body mass index (BMI) was 27.0 ± 5.1 kg/m^2^ (range, 10.2 to 98.0 kg/m^2^). The most common primary indication for THA was osteoarthritis (82.8% [150,845 of 182,118]). Patient demographic characteristics are summarized in Table 1.

**Table 1:** Demographic Characteristics in the Final Data Set (N = 182,118 THAs)*

Parameter	No.	Value†
Age *(yr)*	182,116	68.9 (12-108)
Age category		
<65 yr	60,171	33.0%
65-74 yr	58,831	32.3%
75-84 yr	50,393	27.7%
≥85 yr	12,721	7.0%
NA	2	
Sex		
Female	96,669	53.1%
Male	85,449	46.9%
NA	0	
BMI *(kg/m*^*2*^*)*	158,281	27.0 (10.2-98.0)
BMI category in kg/m^2^		
<18.5	3,228	2.0%
18.5-24.9	56,655	35.7%
25-29.9	60,314	38.0%
30-34.9	27,716	17.5%
35-39.9	8,152	5.1%
≥40	2,756	1.7%
NA	23,297	
ASA category		
1	20,609	12.1%
2	101,121	59.2%
3-5	48,991	28.7%
NA	11,397	
Surgical approach		
Posterior	24,194	13.3%
Anterior	92,868	51.0%
Anterolateral	55,211	30.3%
Lateral	8,483	4.7%
Other approach	1,285	0.7%
NA	77	
Primary indication		
Osteoarthritis	150,845	82.8%
Femoral neck fracture	13,859	7.6%
Osteonecrosis	9,280	5.1%
Other cause	8,134	4.5%
NA	0	
Femoral stem design		
Cementless		
1B	25,172	13.8%
1C	701	0.4%
1D	14,667	8.1%
2	15,797	8.7%
3	82,728	45.4%
4A	224	0.1%
4B	10,046	5.5%
5A	215	0.1%
6A	22	0.01%
6B	1	0.001%
7	4,140	2.3%
Cemented		
Composite-beam	25,686	14.1%
Custom	156	0.09%
Double-tapered	416	0.2%
Triple-tapered	2,147	1.2%
NA	0	

* THA = total hip arthroplasty, BMI = body mass index, ASA = American Society of Anesthesiologists, and NA = not available.

† The values are given as the percentage or the mean with the range in parentheses.

### Outcome

The primary objective of this study was to assess the association between femoral stem geometry and the risk of PFF following primary THA. PFF events were identified through subsequent procedures following the index THA, including revision surgeries and osteosynthesis to treat PFFs without stem revision. The secondary objective was to evaluate risk factors for PFF specifically among patients undergoing elective primary THA.

### Variables

Implant-related variables included the overall fixation type (cemented or cementless), use of a single- or dual-mobility cup, the presence or absence of a collar on the femoral stem, use of a lateralized stem, femoral head size, and the bearing-surface combination. Cementless stem designs were categorized according to the Kheir classification^22^, which divides stems into 7 distinct types on the basis of stem geometry, fixation level (metaphyseal versus diaphyseal), and modularity (Fig. 1; see also Appendix Table 1). Cemented stem designs were categorized as double-tapered polished, triple-tapered polished, composite-beam, or custom.

**Fig. 1:**
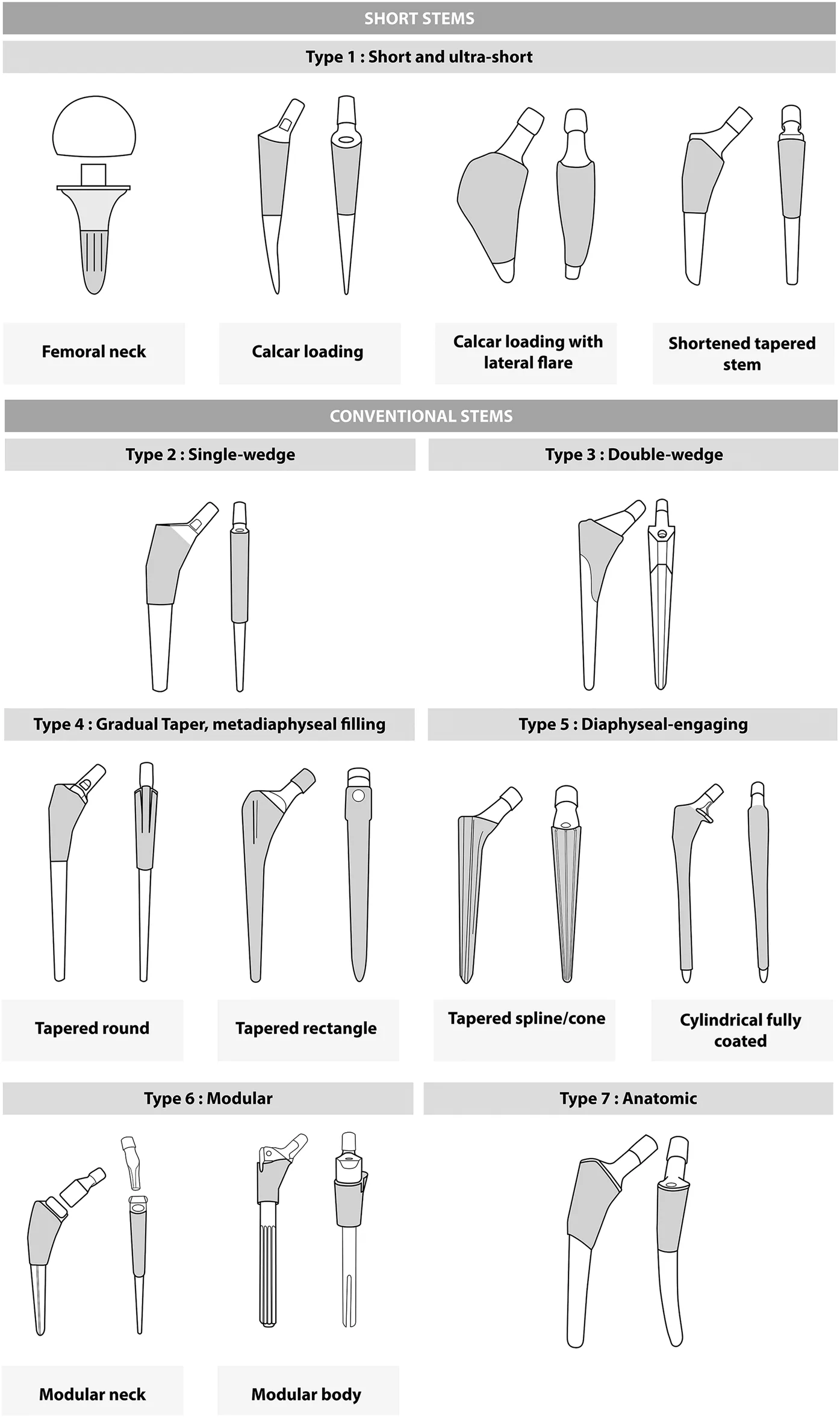
Kheir classification of cementless femoral stem designs. (Based on: Kheir MM, Drayer NJ, Chen AF. An Update on Cementless Femoral Fixation in Total Hip Arthroplasty. J Bone Joint Surg Am. 2020 Sep 16;102(18):1646-61.)

### Statistical Analysis

To identify potential risk factors for PFF, we used a multivariable Cox regression model (number of events available for analysis = 1,226; cause-specific method). Hazard ratios (HRs) are reported with 95% confidence intervals (CIs). For all analyses, a p value of <0.05 (2-sided) was considered significant. All analyses were performed using R Statistical Software (v4.4.2; R Core Team, 2021).

## Results

### Univariate Analysis by Stem Type

The cumulative incidence of PFF was 0.7% at 5 years and 1.3% at 10 years (see Appendix Fig. 1). Revisions were performed as the treatment in 74.7% of cases and osteosynthesis alone, for 25.3%. In univariate analysis (type-3 cementless stems as the reference), the risk of PFF was higher for type-7 (cementless anatomic) (HR = 2.11 [95% CI = 1.62 to 2.74]) and type-1C (cementless short, calcar-loading with lateral flare) stems (HR = 1.98 [95% CI = 1.03 to 3.83]), and lower for triple-tapered cemented (HR = 0.35 [95% CI = 0.14 to 0.83]) and type-1B (cementless short, calcar-loading) stems (HR = 0.76 [95% CI = 0.63 to 0.92]). Full results by stem type are presented in Table 2.

**Table 2:** Univariate Cox Regression According to Stem Type*

Stem Design	HR [95% CI]	P Value
Cementless		
3	1	—
1B	0.76 [0.63; 0.92]	**<0.01**
1C	1.98 [1.03; 3.83]	**0.04**
1D	0.92 [0.74; 1.14]	0.46
2	1.00 [0.82; 1.22]	0.98
4A	0.64 [0.09; 4.52]	0.65
4B	0.86 [0.67; 1.10]	0.23
5A	1.34 [0.33; 5.37]	0.68
6A	0.00 [0.00; inf.]	0.98
6B	0.00 [0.00; inf.]	1.00
7	2.11 [1.62; 2.74]	**<0.01**
Cemented		
Composite-beam	1.01 [0.85; 1.20]	0.90
Custom	2.01 [0.50; 8.05]	0.33
Double-tapered	0.83 [0.21; 3.33]	0.79
Triple-tapered	0.35 [0.14; 0.83]	**0.02**

* Number of cases included in the model = 182,118. HR = hazard ratio, CI = confidence interval, and inf. = infinity.

### Multivariable Analysis

Regarding implant design, stem classification remained a significant predictor of PFF: using stem type 3 as the reference category, cementless type-7 (anatomic) stems were associated with increased fracture risk (HR = 1.88 [95% CI = 1.34 to 2.65]; p < 0.01), whereas cementless type-1B (short, calcar-loading) (HR = 0.62 [95% CI = 0.50 to 0.78]; p < 0.01) and composite-beam cemented (HR = 0.45 [95% CI = 0.36 to 0.55]; p < 0.01) stems were protective. Neither bearing surface nor head size showed a significant association with PFF risk. Stems with a collar conferred strong protection (HR = 0.26 [95% CI = 0.20 to 0.35]; p < 0.01), while use of a dual-mobility cup was independently associated with increased risk (HR = 1.56 [95% CI = 1.18 to 2.07]; p < 0.01).

Compared with patients 65 to 74 years of age, those younger than 65 had a significantly lower risk of PFF (HR = 0.58 [95% CI = 0.48 to 0.71]; p < 0.01), whereas patients 75 to 84 years of age (HR = 1.68 [95% CI = 1.44 to 1.96]; p < 0.01) and ≥85 years of age (HR = 1.86 [95% CI = 1.47 to 2.35]; p < 0.01) had an elevated risk. American Society of Anesthesiologists (ASA) class also influenced fracture risk: compared with male patients with an ASA class of 2, female patients with an ASA class of 2 (HR = 1.41 [95% CI = 1.16 to 1.70]; p < 0.01) or ASA class of 3 to 5 (HR = 1.70 [95% CI = 1.37 to 2.11]; p < 0.01) had a higher risk of PFF, as did male patients with an ASA class of 3 to 5 (HR = 1.73 [95% CI = 1.40 to 2.13]; p < 0.01). Conversely, males with an ASA class of 1 had a lower risk of PFF (HR = 0.57 [95% CI = 0.35 to 0.92]; p = 0.02) compared with males with an ASA class of 2.

BMI extremes were associated with increased risk of PFF, with patients classified as underweight (BMI of <18.5 kg/m^2^) (HR = 1.61 [95% CI = 1.17 to 2.23]; p < 0.01) and individuals with morbid obesity (BMI of ≥40 kg/m^2^) (HR = 1.64 [95% CI = 1.08 to 2.48]; p = 0.02) having significantly higher HRs compared with patients of normal weight. Elective procedures were associated with a lower risk of PFF (HR = 0.36 [95% CI = 0.30 to 0.43]; p < 0.01), whereas prior ipsilateral hip surgery was associated with an increased risk (HR = 1.32 [95% CI = 1.01 to 1.72]; p = 0.04).

Complete results for all variables included in the multivariable model are detailed in Table 3 and Figure 2.

**Table 3:** Multivariable Cox Regression Model*

Variable	HR	95% CI	P Value
Age in yr			
65-74	1	—	—
<65	0.58	0.48; 0.71	**<0.01**
75-84	1.68	1.44; 1.96	**<0.01**
≥85	1.86	1.47; 2.35	**<0.01**
Sex and ASA class			
Male, ASA 2	1	—	—
Female, ASA 1	0.72	0.47; 1.1	0.13
Female, ASA 2	1.41	1.16; 1.70	**<0.01**
Female, ASA 3-5	1.70	1.37; 2.11	**<0.01**
Male, ASA 1	0.57	0.35; 0.92	**0.02**
Male, ASA 3-5	1.73	1.40; 2.13	**<0.01**
BMI in kg/m^2^			
18.5-24.9	1	—	—
<18.5	1.61	1.17; 2.23	**<0.01**
25-29.9	0.96	0.83; 1.12	0.62
30-34.9	0.86	0.70; 1.05	0.14
35-39.9	1.14	0.85; 1.53	0.37
≥40	1.64	1.08; 2.48	**0.02**
Elective THA			
No	1	—	—
Yes	0.36	0.30; 0.43	**<0.01**
Prior ipsilateral hip surgery			
No	1	—	—
Yes	1.32	1.01; 1.72	**0.04**
Dual-mobility cup			
No	1	—	—
Yes	1.56	1.18; 2.07	**<0.01**
Cementless cup			
No	1	—	—
Yes	1.25	0.82; 1.91	0.31
Bearing surface			
MoP	1	—	—
CMoXLP	2.55	0.35; 18.70	0.36
CoC	5.92	0.82; 42.82	0.08
CoP	4.66	0.65; 33.65	0.13
CoXLP	4.82	0.67; 34.67	0.12
CMoP	5.77	0.79; 41.94	0.08
MoM	0	0; inf.	0.99
MoXLP	4.35	0.60; 31.38	0.14
Head size, per mm	1.02	0.99; 1.05	0.16
Stem design			
Cementless			
3	1	—	—
1B	0.62	0.50; 0.78	**<0.01**
1C	1.73	0.86; 3.5	0.13
1D	0.84	0.65; 1.09	0.20
2	0.82	0.64; 1.04	0.11
4A	0.99	0.14; 7.03	0.99
4B	0.82	0.62; 1.07	0.14
5A	1.72	0.43; 6.98	0.45
6A	0	0; inf.	0.99
6B		NA; NA	
7	1.88	1.34; 2.65	**<0.01**
Cemented			
Composite-beam	0.45	0.36; 0.55	**<0.01**
Custom		NA; NA	
Double-tapered	0.37	0.09; 1.49	0.16
Triple-tapered	0.85	0.34; 2.12	0.72
Presence of collar			
No	1	—	—
Yes	0.26	0.20; 0.35	**<0.01**
Lateralized stem			
No	1	—	—
Yes	0.88	0.75; 1.04	0.14

* Number of cases included in the model = 182,118. HR = hazard ratio, CI = confidence interval, ASA = American Society of Anesthesiologists, BMI = body mass index, THA = total hip arthroplasty, MoP = metal-on-polyethylene, CMoXLP = ceramicized metal-on-highly-cross-linked polyethylene, CoC = ceramic-on-ceramic, CoP = ceramic-on-polyethylene, CoXLP = ceramic-on-highly-cross-linked polyethylene, CMoP = ceramicized metal-on-polyethylene, MoM = metal-on-metal, MoXLP = metal-on-highly-cross-linked polyethylene, inf. = infinity, and NA = not available (due to the low number of cases with the given stem).

**Fig. 2:**
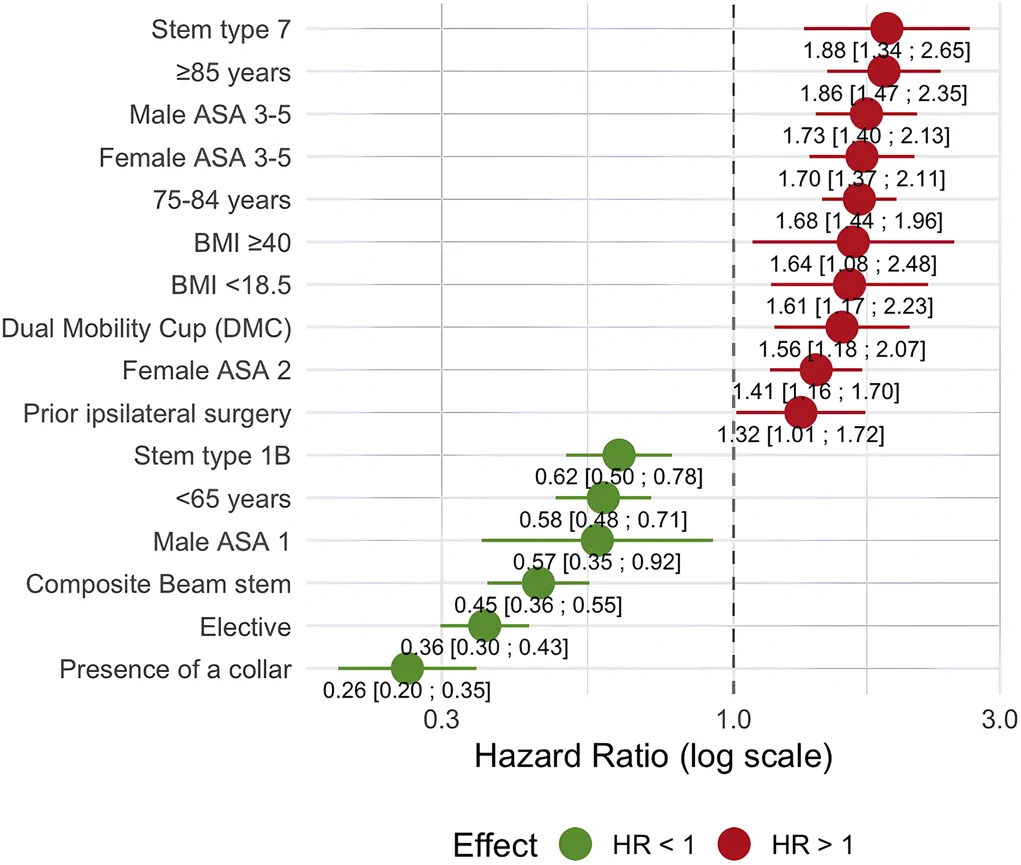
Forest plot illustrating the multivariable-adjusted hazard ratios (HRs) and their 95% confidence intervals for risk factors associated with periprosthetic femoral fracture. Factors with an HR of >1 (shown in red) were associated with an increased risk, whereas factors with an HR of <1 (shown in green) were associated with a protective effect. The HRs are plotted on a logarithmic scale to improve visualization of the effect sizes. ASA = American Society of Anesthesiologists, and BMI = body mass index. The reference groups are given in Table 3.

### Sensitivity Analysis

Using composite-beam cemented stems as the reference, several stem designs showed higher risk of PFF: type 1C (HR = 3.88), 1D (HR = 1.89), 2 (HR = 1.83), 3 (HR = 2.24), 4B (HR = 1.83), and 7 (HR = 4.21). Stratified by indication (elective THA versus THA for fracture), implant-related effects (protective collar, composite-beam stem) remained consistent, whereas age, ASA class, and female sex were stronger predictors in elective THAs. Dual-mobility cups increased risk both in elective (HR = 1.49) and fracture (HR = 1.91) cases, highlighting the predominant role of patient factors in elective surgery and the consistent impact of stem design across indications (see Appendix Fig. 2).

## Discussion

The main finding of this study was the identification of femoral stem design as a significant determinant of PFF risk. Specifically, type-7 stems according to the Kheir classification emerged as a risk factor, whereas composite-beam stems and type-1B stems appeared to be protective. Beyond stem design, other surgical parameters were also identified as influencing fracture risk, notably the presence of a collar (lower risk) and the use of a dual-mobility cup (higher risk).

PFFs are now among the leading causes of revision after THA^10^. This issue is gaining increasing attention, with several studies already demonstrating the influence of stem design on fracture risk^13,23,24^. Beyond fixation method (cemented versus cementless), stem design itself appears to play a critical role^25,26^. Many studies have shown that cemented stems may offer a protective effect^27^. For example, in a study reported by Adik et al.^28^, the incidence of PFFs dropped from 3.5% to 1.7% between 2018 and 2022, which was concurrent with a 2-fold increase in the use of cemented stems among patients >75 years of age.

However, even among cemented stems, design remains crucial. Palan et al.^29^ compared 4 cemented stem models (CPT [Zimmer], C-stem [DePuy International], Charnley [DePuy International], and Exeter V40 [Stryker]), adjusting for age, sex, and ASA class, and found that the CPT had a higher risk of revision for PFF (revision risk ratio of 3.89 [95% CI = 3.07 to 4.93]), while the Charnley had a lower risk (0.41 [95% CI = 0.24 to 0.70]), compared with the Exeter. Similarly, using data from the Swedish Hip Arthroplasty Register to evaluate 2 cemented tapered slip stems, Chatziagorou et al.^30^ found that the Exeter stem had a 10-fold higher risk of type-B fractures compared with the Lubinus SPII stem (Waldemar Link), with no significant difference found for type-C fractures. Notably, the cemented Lubinus SPII stem has a collar, which could play a role.

Similar findings highlighting the importance of stem design have been reported in several joint registry studies. Analyzing American Joint Replacement Registry data, Kelly et al.^31^ observed that Kheir type-2 (single-wedge) and type-3 (double-wedge) stems were associated with an increased risk of PFF (HR = 2.9 and 3.1, respectively; p < 0.001) compared with type-4 stems (taper/metadiaphyseal-filling). Using the U.K. National Joint Registry, Lamb et al.^14^ found that composite-beam stems had the lowest PFF rates, and cementless stems with collars performed similarly to or better than tapered slip stems, confirming that femoral stem design is a key determinant of fracture risk.

Our results clearly showed that type-7 stems were associated with an increased fracture risk, whereas composite-beam and type-1B stems appeared to be protective. These findings align with those of Cottino et al.^32^, who reported that anatomic stems were a risk factor (odds ratio [OR] = 2.2; p < 0.001) compared with straight stems. One possible explanation involves the distribution of bone-mineral density following THA, which varies by femoral stem design^33^. Anatomic stems tend to achieve better proximal canal fill but less central and distal fill, which may alter stress-shielding and weaken the bone over time^34-36^.

Our study also confirms the protective role of the femoral stem collar^37-39^, an effect consistently reported in the literature. For example, in a series of uncemented stems, Favroul et al.^19^ found that the absence of a collar was the only factor significantly associated with a higher fracture risk (OR = 7.22 [95% CI = 1.02 to 51.28]; p = 0.048). Similarly, Kelly et al.^31^ found that the absence of a collar significantly increased fracture risk (HR = 7.8 [95% CI = 4.1 to 15]; p < 0.001). Supporting these findings, Giovanoulis et al.^40^, in a systematic review, reported a significant reduction in fracture risk for stems with collars (relative risk [RR] = 0.52 [95% CI = 0.29 to 0.92]; p = 0.03). A biomechanical explanation was provided by Lamb et al.^41^, who noted that collarless stems tend to first deform the trabecular bone before engaging the cortex, thus increasing the risk of calcar fractures. In contrast, collars rest directly on the calcar, promoting load distribution and enhancing rotational stability.

Finally, our study identified an association between the use of a dual-mobility cup and PFF risk, an observation not previously described, to our knowledge. While we initially considered the possibility of confounding by factors such as age, surgical indication, or stem type, these variables were accounted for in the multivariable model. One hypothesis is that the increased jump distance with dual-mobility constructs leads to higher torsional forces on the femoral stem. Alternatively, debris from non-highly-cross-linked polyethylene may contribute to metaphyseal osteolysis, thereby weakening the bone. This finding warrants further study to elucidate the underlying mechanism.

This study had several limitations. First, certain stem categories in the Kheir classification were not equally represented. However, this is the first comprehensive analysis of its kind, and the stems most frequently used in our study correspond to the most widely used models in other national registries, supporting external validity. In addition, PFFs treated nonoperatively (mainly Vancouver A^42^) are not captured in the SIRIS registry; however, these fractures are generally less influenced by stem design, except for those sustained intraoperatively. Other risk factors identified in our study, such as female sex, older age, low and high BMI, and greater ASA class, are consistent with the literature, supporting the representativeness of our cohort^18,43,44^. Race and ethnicity data are not collected in the SIRIS registry. The absence of these variables represents a limitation that may affect the generalizability of the study findings. Moreover, residual confounding may partly explain the observed higher risk of PFF with dual-mobility cups, as patients receiving these implants often have additional risk factors for falls and prosthesis dislocation (e.g., cognitive impairment, neurological diseases) that are not fully captured in the registry. Lastly, we did not account for all potential interactions between femoral and acetabular implant designs; however, the multivariable model included fixation type and the use of a dual-mobility cup, which helps limit this bias.

In conclusion, our results confirm the critical role of femoral stem design and the presence of a collar in reducing PFF risk. Anatomic stems were associated with higher risk, whereas composite-beam cemented stems and collared designs were protective. These findings also highlight the importance of individualized implant selection, taking into account patient-specific factors, especially for older patients or those with frailty.

## Appendix

Supporting material provided by the authors is posted with the online version of this article as a data supplement at jbjs.org (http://links.lww.com/JBJS/J111).
